# Clinical response in patients with ovarian cancer treated with metronomic chemotherapy

**DOI:** 10.3332/ecancer.2017.723

**Published:** 2017-02-28

**Authors:** Herman Andrés Perroud, O Graciela Scharovsky, Viviana Rosa Rozados, Carlos María Alasino

**Affiliations:** 1 Experimental Oncology Section, Institute of Experimental Genetics, School of Medical Sciences, National University of Rosario, Rosario 2000, Argentina; 2 National Scientific and Technical Research Council (CONICET), Rosario 2000, Argentina.; 3 Italian Hospital of Rosario, Department of Clinical Oncology, Rosario 2000, Argentina; 4 Research Council of the National University of Rosario (CIUNR), Rosario 2000, Argentina; 5 Institute of Oncology of Rosario, Rosario 2000, Argentina

**Keywords:** ovarian cancer, metronomic chemotherapy, cyclophosphamide

## Abstract

Ovarian cancer (OC) is the leading cause of death from gynaecological cancer. It is extremely hard to diagnose in the early stages and around 70% of patients present with advanced disease. Metronomic chemotherapy (MCT) is described as the chronic administration of, generally low, equally spaced, doses of chemotherapeutic drugs with therapeutic efficacy and low toxicity. This is an effective and low-cost way to treat several types of tumours, including ovarian cancer. Here, we present six cases of advanced ovarian cancer treated with MCT with low doses of cyclophosphamide, which showed clinical response and stable disease.

## Introduction

Ovarian cancer (OC) is the leading cause of death from gynaecological cancer in the United States, and accounts for 5% of cancer deaths among women, causing more deaths than any other gynaecological cancer. Unfortunately, less than 40% of women with ovarian cancer are cured [1]. In Argentina, OC is the fifth most common cancer in women, with approximately 2300 new cases per year, and it represents the sixth leading cause of cancer mortality in women [2]. The incidence of OC increases with age and it is higher in the sixth and seventh decade of life [3].

OC is extremely hard to diagnose in the early stages [4]. While most of the patients who are diagnosed with ovarian cancer at the early stages are asymptomatic, when clinical symptoms appear, patients have already developed middle- to late-stage tumours. Around 70% of patients present with advanced disease; the stage of the tumour when it is diagnosed as extremely important, because the 5-year survival is about 92% for the early stages, while it decreases to 18% for stage IV [5].

For non-metastatic OC, surgery remains the most important treatment, followed or not by adjuvant treatment with intravenous (IV) or intraperitoneal (IP) chemotherapy with platinum and taxanes [6–8]. The overall survival achieved with IP paclitaxel/cisplatin is longer than that obtained with IV paclitaxel/cisplatin in patients with optimal cytoreductive surgery [9]. Schemes with carboplatin have been demonstrated to be effective in patients with poor performance status [10].

Other treatment regimens for OC have been proposed, like hormone treatment [11], or targeted therapies using bevacizumab. However, the benefits of bevacizumab treatment are in question [12].

Low-dose metronomic chemotherapy (MCT) described as the administration of chronic, equally spaced and, generally, low doses of chemotherapeutic drugs without extended rest periods, that allows chronic treatment with therapeutic efficacy and low toxicity, has been demonstrated to be effective in several types of tumours (breast, sarcoma, lung, prostate), the decrease in vascular endothelial growth factor being one of the demonstrated mechanisms of action [13–16], which results in an antiangiogenic effect. Besides, MCT has been proved to modulate the host immune response; this effect is important considering that the anticancer immune response may be crucial for the long-term control of cancer. MCT can tilt the balance from immunosuppression to immunostimulation by several mechanisms, such as induction of immunogenic cancer cell death, enhancement of antigen presentation by dendritic cells, increase in cancer cells immunogenicity, depletion of regulatory T cells and modulation of myeloid-derived suppressor cells, enhancement of the cytotoxic activity of immune effector cells like tumour-specific T cells and γδT cells [17]. Moreover, since OC is strongly immunogenic, the therapeutic utility of immune checkpoint inhibitors, which would be able to overcome the tumour immune escape, is being explored [18]. Regulatory T cells (Tregs) is consider a poor prognosis factor. Interestingly, low doses of Cy administered metronomically have shown to decrease the level of Treg cells in tumour infiltrates [19, 20].

Another benefit described for MCT is the improvement of patients´ quality of life (QOL) or, at least, the absence of adverse changes in QOL [21].

At present, there are not many MCT trials involving OC, and most of the literature deals with case reports, or retrospective studies. In this case series report, we describe the evolution of six patients with advanced OC treated with metronomic cyclophosphamide as a palliative treatment.

## Methods and case report

Six patients from the Italian Hospital of Rosario with advanced metastatic disease, refractory to standard chemotherapy, who were treated with metronomic cyclophosphamide were reviewed. The cases herein reported are consecutive and the total number of OC patients treated with MCT between 2012 and 2016 in the Italian Hospital of Rosario. The treatment selection was according to the attending doctor decision. Responses were evaluated using RESIST 1.1 and disease control was done using CT scan or MRI. Table 1 shows a summary of the main findings for each patient. Table 2 shows the treatments received and the PFS for each one.

**Figure 1.**
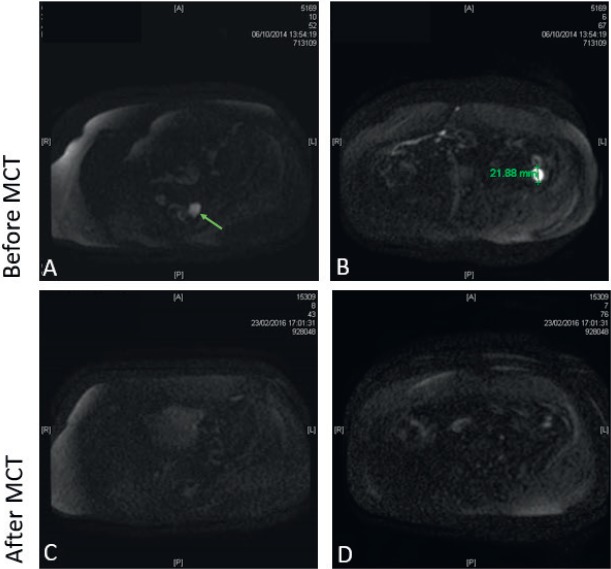
Pelvic magnetic resonance imaging using diffusion-weighted image. (A) Solid tumour lesion (arrow) of 2 cm pararectal space left rear; (B) a 2.2 cm tumour lesion in left paracolic pit. Both with enhancement in the diffusion restriction sequences; (C and D) after MCT, there are no signs of those lesions.

**Figure 2.**
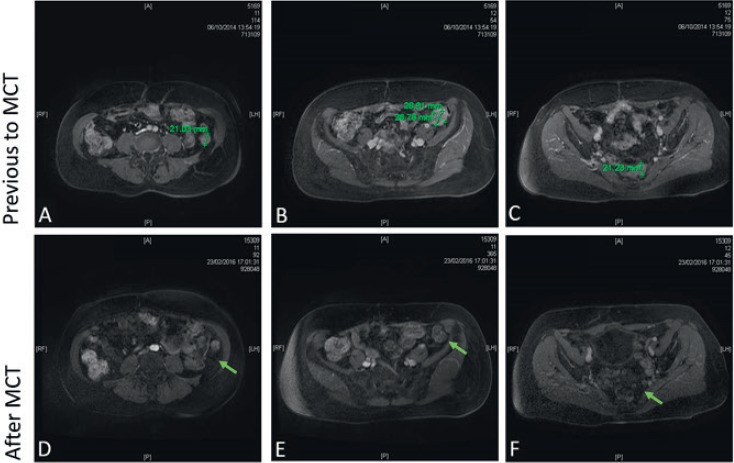
Contrast-enhanced pelvic magnetic resonance imaging. Previous MCT: (A) a 2.1 cm solid tumour in left paracolic grave, the tumour appears in close association with the rear edge of descendent colon and the left latero-conus fasia; (B) a 2.6 cm × 2.8 cm solid tumour lesion in contact with sigmoid colon; (C) a 2 cm tumour lesion in the pararectal space left rear. A, B, and C showed contrast enhancement after intravenous administration of gadolinium. After MCT: (D) no lesion in the left paracolic grave (arrow); (E) residual tumour lesion without diffuse heterogeneous enhancement with intravenous administration of gadolinium (arrow); (E) no lesion observed in the pararectal space (arrow).

**Patient 1:** A 63-year-old female, with no background of serious diseases, who consulted for pain, abdominal distention, and ascites, was diagnosed a stage IIIC poorly differentiated ovarian endometrioid adenocarcinoma in July 2011. The preoperative level of cancer antigen-125 (CA125) was 200.6 U/ml. The surgeon performed a R0 resection and then she received standard chemotherapy. After relapse, and due to the patient condition, low doses of cyclophosphamide (Cy) were considered. She received MCT with Cy (50 mg per oral, once daily -p.o.d-) since October 2014 and she is still on treatment; CA125 levels began a slow decrease, reaching normal values after 4 months of treatment. After 17 months of MCT a control MRI showed a major partial response (Figures 1 and 2). The patient is still under low doses of Cy, with good tolerance to medication and good QOL.

**Patient 2:** A 55-year-old female was diagnosed with bulky stage IIIC ovarian endometrioid adenocarcinoma. Pre-operative CA-125 was 114 U/ml. The patient was treated with neo adjuvant chemotherapy and then, R0 resection surgery. Due to a significant decrease in her QOL in the fourth relapse (3 lines of chemotherapy and hormonal treatment) she started MCT with Cy (50 mg p.o.d). CA125 levels decreased from 55 U/ml to 9 U/ml in 5 months. Treatment was well tolerated with clinical improvement and weight gain. She received MCT from February 2014 to February 2016. After almost 24 months of treatment with stable disease, the patient progressed and she was switched to topotecan+bevacizumab, with very bad tolerance, deterioration of QOL and without therapeutic response. The patient is still alive in palliative care at the moment.

**Patient 3:** A 57-year-old woman was diagnosed with a stage IIIC epithelial ovarian cancer. Pre-operative CT showed ascites and abdominal mass. Pre-operative CA125 was 800 U/ml; she underwent optimal cytoreductive surgery and adjuvant chemotherapy. Due to progression, she received four lines of chemotherapy before MCT. She began metronomic Cy (50 mg p.o.d) after increase in the size of hepatic metastasis. CA125 decreased without reaching normal levels (values from 45 to 60 UI/ml). The patient recovered from the toxicity of the last chemotherapy treatment, kept working and had a normal way of living. Transient Cy dose reductions were indicated, due to G1 haematological toxicities. CT scans showed stable disease. She received MCT from March 2012 to November 2013. Progression was detected after 20 months of treatment. The patient was lost to follow-up.

**Patient 4:** A 56-year-old female, with no background of serious diseases, was diagnosed stage IIIC poorly differentiated infiltrating papillary adenocarcinoma of the ovary. Surgical exploration confirmed ascites and positive peritoneal implants. R0 Resection surgery was performed. Pre-operative level of CA125 was 16,860.6 U/ml. She received initial standard chemotherapy with early recurrence and two additional lines of chemotherapy, showing minor responses and rapid progression. With an inoperable pelvic mass and poor performance status, she started MCT (Cy, 50 mg p.o.d). The treatment elapsed with minimal toxicity, and ascites was controlled with diuretics. The patient’s condition remained stable with ECOG 2 for 6 months, after which she worsened gradually and died because of pleural effusion and lung metastasis.

**Patient 5:** A 52-year-old female, with a background of hypothyroidism, presented with abdominal distention and a pelvic tumour in a CT scan. She was diagnosed with a stage IIIC infiltrating endometrioid ovarian adenocarcinoma. She received surgery and standard chemotherapy with a total of three lines of treatments. After progression, she started MCT Cy (50 mg p.o.d) with a minimal hematological toxicity. MCT began in September 2014 with no interruptions and, at present, the patient continues the treatment. The patient’s condition remained stable with good QOL (ECOG 1). The patient is still alive (June 2016) with 21 months of PFS at the moment of manuscript writing.

**Patient 6:** A 54-year-old woman was diagnosed with a stage IIIC papillary ovarian adenocarcinoma. Pre-operative CA125: 1648 U/ml; she underwent optimal cytoreductive surgery and received adjuvant chemotherapy. She received two lines of chemotherapy, a new surgery of the peritoneal masses and hormonal treatment before MCT. She was treated with MCT (Cy 50 mg p.o.d) with good tolerance and stable disease for 12 months. She received MCT from December 2013 to December 2014. After that period, the tumour progressed and she began a scheme with targeted therapy (bevacizumab) plus chemotherapy with partial response, severe toxicity and rapid progression. The patient is still alive and is under liposomal doxorubicin (LSD) chemotherapy at the moment of manuscript writing.

## Discussion

Here, we present six patients with metastatic OC in which metronomic Cy provided prolonged stable disease, clinical benefit, and one case with near clinical complete response.

The use of platins and taxanes has improved the overall survival for OC. The outcomes depend not only on the chemotherapy schemes, but also on others factors like response to first line chemotherapy, disease free survival upon surgery, and number of previous treatments [22].

Metronomic chemotherapy with low doses of Cy has been proposed as a potential treatment of OC due to its antiangiogenic and immunomodulating properties [17]. However, the clinical trials that support this approach are scarce, and most of the information comes from case reports [23] and pre-clinical studies. Merritt *et al*., in a OC pre-clinical model, described that metronomic topotecan has antiproliferative effect and targets some pro-angiogenic mediators like Hif-1 alfa, suggesting it as a novel therapeutic strategy [24]. Likewise, the use of nabpaclitaxel and metronomic topotecan showed significant reductions in tumour weight and proliferation as well as pazopanib did. Those agents seem to be ideal for clinical trials [25, 26]. On the other hand, regarding the resistance to the drugs administered metronomically in different types of tumours, including OC, Chow *et al* suggested that the sensitivity to MCT using different drugs do not only depends on the antiangiogenic mechanism, but also on other unknown additional mechanisms that could be drug-specific [27].

One of the most controversial topics in OC is the use of antiangiogenic/target drugs. The combination of bevacizumab with carboplatin/paclitaxel is associated with longer disease-free survival than the combination alone. But no significant difference has been observed in terms of overall survival rate [28]. Also, the use of bevacizumab as a maintenance drug subsequent to chemotherapy has not offered benefits in survival, resulting in the addition of many side effects, and the increase in the treatment costs [29]. Since the evidence shows that bevacizumab does not appear to confer any survival advantage, the National Comprehensive Cancer Network panel does not recommend its use.

In the clinical field, two retrospective studies analysed the role of MCT in OC. Sánchez-Muñoz *et al* suggested that the combination of metronomic oral Cy plus bevacizumab in heavily pretreated patients with recurrent OC could be an option, with a response rate of 40% and a Clinical Benefit of 48% [30]. Also, Ferrandina *et al* showed the efficacy and safety of metronomic oral Cy in heavily treated relapsed OC; the patients responding to MCT showed higher PFS and OS compared to those who had stable disease or disease progression [31]. Moreover, a case report by Samaritani *et al* presented a patient with stage-IIIC OC treated with metronomic Cy, who showed an impressive PFS of 65 months, without side effects, after non-response to standard chemotherapy with platins, taxans, and topotecan [23]. Those results are very encouraging and the prolonged PFS accomplished agrees with the ones herein described. The non-randomized clinical trial conducted by El-husseiny *et al*, setting metronomic Cy and methotrexate as maintenance therapy in patients who achieved complete response after platinum and paclitaxel regimens, showed that the PFS was significantly higher (18 months) in the treatment arm than that of the observational one (15 months). Also, no grade III–IV toxicities were observed and the treatment regimen was safe and well tolerated [32]. Furthermore, the addition of bevacizumab (10 mg/kg) IV every two weeks to metronomic oral Cy 50 mg/d in recurrent OC showed a median PFS of 7.2 months, with mild toxicity [33].

It is well known that angiogenesis plays an important role in tumour growth and dissemination, and it is a key process in OC progression [15]. However, its intended inhibition with bevacizumab has not achieved the main objective, neither for adjuvant nor for maintenance or metastatic settings, causing serious side effects and increasing the costs of patient care. Some single and series case reports described the benefit of adding bevacizumab to MCT with Cy [34–36]; and one retrospective review describe that the addition of Cy to intravenous bevacizumab in heavily pre-treated patients with recurrent ovarian carcinoma improved PFS and OS in responders. However, the adverse events are not well described [37, 38]. Also, the cost-effective ratio of bevacizumab in OC is not totally clear [39], as well as the combination of bevacizumab with metronomic Cy. Hence, well designed clinical protocols are needed to clarify this matter.

One important aspect to highlight is that MCT did not add toxicity in our patients, or did not worsen the QOL. Unfortunately, along with the scarcity of clinical trials of MCT for OC, there are no objective evaluations concerning QOL. As mentioned before, platin and taxanes have been intensively used in the treatment of ovarian cancer. Interestingly a study population using metronomic weekly paclitaxel plus carboplatin showed to be effective and well tolerated in those patients who did not tolerate the standard 21-day treatment [40].

Our results in this cohort of patients showed that the median PFS obtained with MCT-based chemotherapy (16.6 months) was slightly higher than that obtained with all of the previous standard treatments administered to those patients (13.3 months), with the advantage of low toxicity and its positive influence on QOL, suggesting that MCT could be utilized as an early treatment option for patients with recurrent disease.

On the other hand, patients with endometrioid adenocarcinomas (3/6) showed a higher mean PFS (20.6 months) than that of patients with papillary adenocarcinoma (12.6 months -3/6), both in the metronomic-based chemotherapy, suggesting that the antiangiogenic properties of MCT would be more specific and sensitive for the endometrioid type of OC. Bamberger *et al* sustained that VEGF could be a useful marker of angiogenesis and tumour progression and that it is involved in ascites formation. Also, they affirm that, in spite the fact that tumour microvessel density is a prognostic factor in various types of cancer, apparently, in OC there is no correlation between that density and age, tumour size, ascites, or tumour growth [41]. Furthermore, up to this moment, there are no studies on the effect of MCT in different types of OC.

In conclusion, OC represents a challenge for medical oncologists, in part for the high frequency of advanced disease at diagnosis, and also because of the lack of better systemic treatment approaches. Surgery, platins, and taxanes represent the keystones in the benefits for survival. Other chemotherapeutics schedules are toxics and did not demonstrate overall survival benefits. The use of old approved non-anticancer drugs for treating cancer is an attractive strategy to offer the patients more therapeutic options. The concept behind this strategy was developed by researchers integrating the Repurposing drugs in oncology (ReDO) Project [42]. Metronomics, a term coined by N. André *et al* refers to “all anticancer treatment regimens combining metronomic chemotherapy and drug repositioning”. Metronomics has gained importance, mainly for healthcare systems with budgets that do not support high-cost new oncology drugs [43, 44]. Studies utilising metformin [45], itraconazole [46], celecoxib [13], propranolol [47], clarithromycin [48], etc. have demonstrated the validity and potential of this therapeutic approach.

## Conclusions

In summary, based on the clinical responses obtained and the low toxicity of the treatment, metronomic chemotherapy and drug repurposing are options to be taken into account for OC therapeutics. Clinical trials are needed to corroborate these findings.

- The treatment with low doses of cyclophosphamide resulted in therapeutic response which varied from near clinical complete response to long stable disease.
- Metronomic chemotherapy could be a feasible treatment option for ovarian cancer patients.
- Metronomic chemotherapy with oral cyclophosphamide presented a low toxicity profile and did not affect the patient’s quality of life.

## Funding

This work was supported by Roemmers Foundation and Ministry of Science, Technology and Innovation of Santa Fe, Argentine. HAP and OGS are fellows of National Scientific and Technologic Research Council (CONICET).

## Ethical standards

The authors declare that the protocol herein described complies with the current laws of Argentina. The protocol was authorised by the Bioethics Committee of the School of Medical Sciences, National University of Rosario.

## Conflict of interest statement

The authors declare no potential conflict of interest.

## Figures and Tables

**Table 1.** Patient characteristics summary.

	Patient 1	Patient 2	Patient 3	Patient 4	Patient 5	Patient 6
**Age at diagnosis (years)**	63	55	57	56	52	54
**Age at MCT (years)**	66	63	63	59	56	57
**Diagnosis**	Endometrioid adenocarcinoma	Endometrioid adenocarcinoma	Papillary adenocarcinoma	Papillary adenocarcinoma	Endometrioid adenocarcinoma	Papillary adenocarcinoma
**Stage at diagnosis**	IIIC	IIIC	IIIC	IIIC	IIIC	IIIC
**Stage at MCT**	IIIC	IV	IV	IIIC	IV	IV
**No of previous lines of chemotherapy**	2	3	4	3	3	2
**IP/HIPEC**	No/Yes	No/No	No/No	Yes/No	No/No	Yes/No
**Hormone therapy**	No	Yes	Yes	No	No	Yes
**Metastatic sites**	Intra-abdominal	Lung, liver	Lung, liver	Pelvic, regional	Hepatic, pelvic	Hepatic
Mean PFS (months)*	13.5	24,5	18	6	7	11
**PFS with MCT (months)**	17	24	20	6	21	12
**Best clinical response with MCT**	nPCR	SD	SD	SD	SD	SD
**Performance Status (ECOG) prior/post MCT**	3/1	2/1	1/1	2/2	1/1	2/2

MCT: metronomic chemotherapy; IP: intra-peritoneal chemotherapy; HIPEC: hyperthermic intra-peritoneal chemotherapy. PFS: progression-free survival.

* mean PFS calculated with the PFS of each line of standard treatment.

**Table 2.** Treatments summary.

	Patient 1	Patient 2	Patient 3	Patient 4	Patient 5	Patient 6
**First line of treatment**	C + P IV × 6 cycles	C + P IV × 6 cycles	C + P IV × 6 cycles	C + P IP × 6 cycles*	C + P IV × 6 cycles	C + P IV × 6 cycles
**PFS (months)**	16	37	26	4	14	19
**Second line of treatment**	C + P IV × 6 cycles + HIPEC	Tamoxifen	C + P IV × 6 cycles + Tamoxifen	LSD	Carboplatin monodrug **	Cisplatin IP
**PFS (months)**	11	36	24	6.6	2	9
**Third line of treatment**	MCT Cy 50 mg p.o.d	C + P IV × 6 cycles	Carboplatin monodrug	Carboplatin monodrug	LSD	Tamoxifen
**PFS (months)**	17 (OG)	12	11	7	5	5
**Fourth line of treatment**		LSD	LSD	MCT Cy 50 mg p.o.d	MCT Cy 50 mg p.o.d	MCT Cy 50 mg p.o.d
**PFS (months)**		13	10	6	21 (OG)	12
**Fifth line of treatment**		MCT Cy 50 mg p.o.d	MCT Cy 50 mg p.o.d			Carboplatin, gemcitabine + bevacizumab
**PFS (Months)**		24	20			N/A
**Sixth line of treatment**		Topotecan + Bevacizumab				LSD
**PFS (Months)**		2				N/A

* Patients treatment with IV schemes finished due to complications with the port.

** Patient presented severe anaphylactic reaction, and treatment was stopped.

OG: on going. C+P: carboplatin + paclitaxel; MCT: metronomic chemotherapy; LSD: liposomal doxorubicin; N/A: not available.
